# Chromosome-scale genome sequencing, assembly and annotation of six genomes from subfamily *Leishmaniinae*

**DOI:** 10.1038/s41597-021-01017-3

**Published:** 2021-09-06

**Authors:** Hatim Almutairi, Michael D. Urbaniak, Michelle D. Bates, Narissara Jariyapan, Godwin Kwakye-Nuako, Vanete Thomaz Soccol, Waleed S. Al-Salem, Rod J. Dillon, Paul A. Bates, Derek Gatherer

**Affiliations:** 1 Division of Biomedical & Life Sciences, Faculty of Health & Medicine, Lancaster University, Lancaster, LA1 4YT UK; 2 Ministry of Health, Riyadh, Saudi Arabia; 3 Department of Parasitology, Faculty of Medicine, Chulalongkorn University, Bangkok, 10330 Thailand; 4 Department of Biomedical Sciences, School of Allied Health Sciences, College of Health & Allied Sciences, University of Cape Coast, Cape Coast, Ghana; 5 Laboratório de Biologia Molecular, Programa de Pós Graduação em Engenharia de Bioprocessos e Biotecnologia, Universidade Federal do Paraná, Curitiba, Brazil

**Keywords:** Parasite genomics, Sequence annotation

## Abstract

We provide the raw and processed data produced during the genome sequencing of isolates from six species of parasites from the sub-family *Leishmaniinae*: *Leishmania martiniquensis* (Thailand), *Leishmania orientalis* (Thailand), *Leishmania enriettii* (Brazil), *Leishmania* sp. Ghana, *Leishmania* sp. Namibia and *Porcisia hertigi* (Panama). *De novo* assembly was performed using Nanopore long reads to construct chromosome backbone scaffolds. We then corrected erroneous base calling by mapping short Illumina paired-end reads onto the initial assembly. Data has been deposited at NCBI as follows: raw sequencing output in the Sequence Read Archive, finished genomes in GenBank, and ancillary data in BioSample and BioProject. Derived data such as quality scoring, SAM files, genome annotations and repeat sequence lists have been deposited in Lancaster University’s electronic data archive with DOIs provided for each item. Our coding workflow has been deposited in GitHub and Zenodo repositories. This data constitutes a resource for the comparative genomics of parasites and for further applications in general and clinical parasitology.

## Abstract (summary)

**Table:** 

Measurement(s)	DNA • genome • sequence_assembly • sequence feature annotation
Technology Type(s)	DNA sequencing • Oxford Nanopore Sequencing • Illumina sequencing • sequence assembly process • sequence annotation
Sample Characteristic - Organism	Leishmaniinae
Sample Characteristic - Location	Namibia • Thailand • Ghana • Brazil

Machine-accessible metadata file describing the reported data: 10.6084/m9.figshare.15134085

## Background & Summary

Leishmaniasis is a neglected tropical disease. It is considered to be a disease of poverty, primarily affecting low and middle-income countries (LMICs). Leishmaniasis is caused by parasites of the genus *Leishmania* and 18 different species are known to infect humans^1^. 98 sandfly species are suspected or confirmed vectors of *Leishmania*^2^. There are three major types of leishmaniasis: visceral, also known as kala-azar, is fatal if left untreated in over 95% of cases; cutaneous, the most common form, causes skin lesions leaving life-long scars and serious disability or stigma; mucocutaneous, leads to partial or total destruction of mucous membranes of the nose, mouth and throat^3^. Over one billion people live in endemic areas and are at risk of leishmaniasis. It is estimated that each year, globally, new cases of cutaneous leishmaniasis occur at an incidence of 700,000 to 1.2 million or more in over 100 countries^4^. Additionally, up to 300,000 visceral leishmaniasis cases cause more than 200,000 deaths annually^5^.

The genus *Leishmania* is divided into four subgenera: *L. Leishmania, L. Viannia, L. Sauroleishmania* and the newest subgenus *L. Mundinia*, the latter now accommodating several species from the *L. enriettii* complex and others, from five continents^6–12^. In 1994, the Leishmania Genome Network was initiated^13^ and announced, ten years later, the assembly of the *Leishmania major* Friedlin strain as the first *Leishmania* reference genome^14^. Since then, a total of 58 genomes have become available publicly, assembled at a variety of levels of completeness ranging from contigs to chromosome level. Prior to our project, only two *L. Mundinia* subgenus genomes have been sequenced and assembled: *Leishmania enriettii*, strain LEM3045 (GCA_000410755) and *Leishmania sp*. MAR, strain LEM2494 (GCA_000410755). The genus *Porcisia* is a sister genus of *Leishmania* within the sub-family *Leishmaniinae*. Prior to the release of our genome, there were no genome sequences for genus *Porcisia*. Subsequently, the partial genome of *P. deanei* was released and published^15^.

We assembled and annotated the genomes of five *L. Mundini*a species – those of *L. martiniquensis*, *L. orientalis*, *L. enriettii*, *L*. sp. Ghana and *L*. sp. Namibia - and one genome in the genus *Porcisia* – that of *P. hertigi*, formerly known as *L. hertigi*^16^ - using Illumina and Nanopore sequencing. The two isolates from Ghana and Namibia are from new species that have not yet been formally named. The World Health Organization (WHO) codes for the six isolates are: *L. martiniquensis* MHOM/TH/2012/LSCM1;LV760; *L. orientalis* MHOM/TH/2014/LSCM4;LV768; *L. enriettii* MCAV/BR/2001/CUR178;LV673; *L*. sp. Ghana MHOM/GH/2012/GH5;LV757; *L*. sp. Namibia MPRO/NA/1975/252;LV425; and *P. hertigi* MCOE/PA/1965/C119;LV43. Nanopore long reads were used for the initial scaffolding assemblies, followed by mapping of the Illumina short reads onto these scaffolds, thus increasing quality of the assembled sequence while preserving whole chromosome integrity. Final polishing, reordering and reorienting of chromosomes, along with masking and classifying of repeat regions, was guided by the most closely related reference genome for each species. Finished genome annotation was both evidence-based and *ab initio*.

**Fig. 1:**
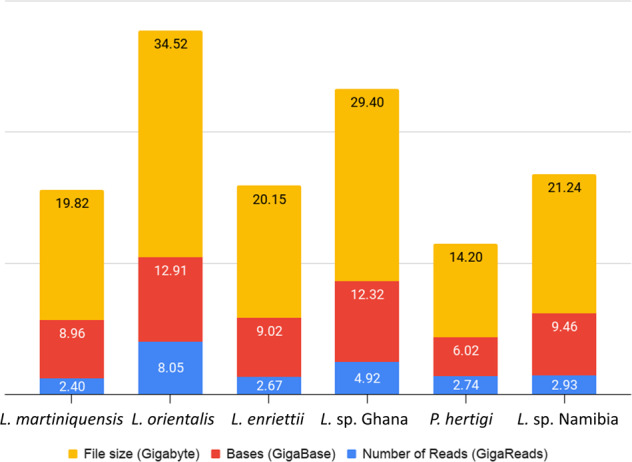
Stacked column chart showing number of sequenced reads in GigaReads (blue), number of yielded bases in GigaBases (red), and the file sizes in Gigabytes (yellow) for each genome assembly.

**Fig. 2:**
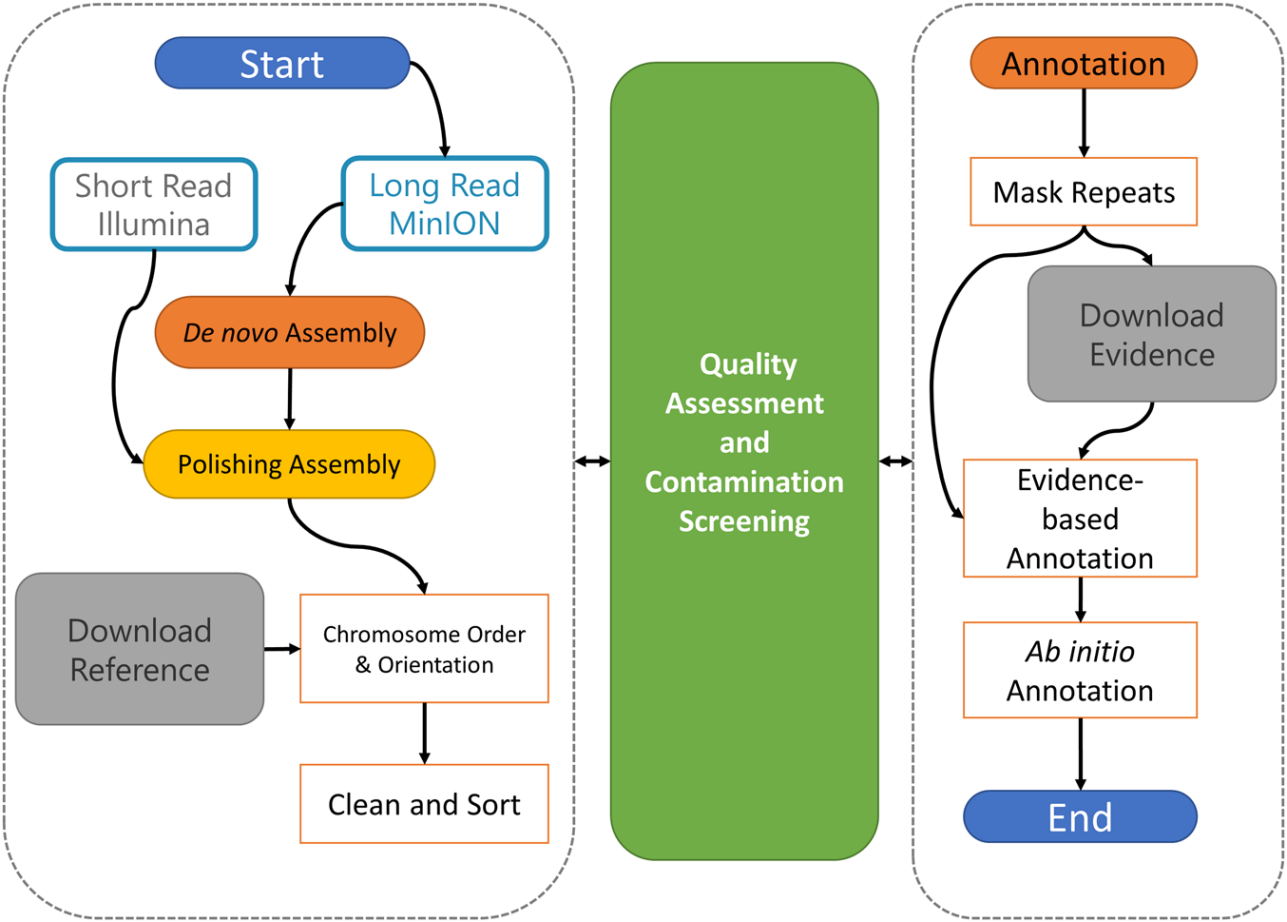
Flowchart showing the analysis workflow strategy.

Figure 1 summarises data sizes and total yield per sample. The total sequencing data file size for all samples was 139.33 Gigabytes, yielding 58.70 GigaBases of sequence data from 23.71 GigaReads. Figure 2 summarises our analysis workflow. This workflow generated four main outputs for each assembly: genome, proteome, and transcriptome files in FASTA format, and a General Feature Format file (GFF) that contains the coordinates for all proteins and transcripts in the assembly.

## Methods

### Sample collection, sequencing and software

From the parasite cryobank at Lancaster University, we selected six samples of the species listed above without publicly available reference genomes. Table 1 gives details for strains, isolates, BioSample and BioProject accessions^17–28^. Illumina HiSeq 4000 and MiSeq sequencing was contracted to BGI Genomics and Aberystwyth University. Nanopore sequencing was performed in-house using MinION FLO-MIN106 flow cells with SQK-LSK109 ligation sequencing protocol. Throughout the text we provide literature citations to software where available. Links to both published and unpublished software used are provided in Table 2. We created public GitHub and Zenodo repositories for the analysis pipeline^29,30^.

**Table 1:** Sample descriptions for all assemblies.

Sample	Strain	Isolate	BioSample	BioProject
*L. (Mundinia) martiniquensis*	LV760	LSCM1	SAMN17294109	PRJNA691531
*L. (Mundinia) orientalis*	LV768	LSCM4	SAMN17294111	PRJNA691532
*L. (Mundinia) enrietti*	LV763	CUR178	SAMN17294112	PRJNA691534
*L. (Mundinia)* sp. Ghana	LV757	GH5	SAMN17294115	PRJNA691536
*L. (Mundinia)* sp. Namibia	LV425	253	SAMN17294129	PRJNA689706
*Porcisia hertigi*	LV43	C119	SAMN17294121	PRJNA691541

**Table 2:** Tools used in analysis workflow with conda or docker link.

Tool	Website	conda or docker link
AGAT	https://github.com/NBISweden/AGAT	https://anaconda.org/conda-forge/agate
AUGUSTUS	http://bioinf.uni-greifswald.de/webaugustus/about	https://hub.docker.com/r/hatimalmutairi/lmgaap-maker
BCFtools	http://samtools.github.io/bcftools/	https://anaconda.org/bioconda/bcftools
bedtools	https://bedtools.readthedocs.io/en/latest/	https://anaconda.org/bioconda/bedtools
blast+	https://blast.ncbi.nlm.nih.gov/Blast.cgi	https://anaconda.org/bioconda/blast
FastQC	https://www.bioinformatics.babraham.ac.uk/projects/fastqc/	https://anaconda.org/bioconda/fastqc
Flye	https://github.com/fenderglass/Flye	https://anaconda.org/bioconda/flye
funannotate	https://github.com/nextgenusfs/funannotate	https://anaconda.org/bioconda/funannotate
GAAS	https://github.com/NBISweden/GAAS	https://anaconda.org/bioconda/gaas
GeneMark	http://exon.gatech.edu/GeneMark/	https://hub.docker.com/r/hatimalmutairi/lmgaap-maker
Genometools	http://genometools.org/	https://anaconda.org/bioconda/genometools-genometools
interproscan	https://www.ebi.ac.uk/interpro/search/sequence/	https://hub.docker.com/r/blaxterlab/interproscan
MAKER2	https://www.yandell-lab.org/software/maker.html	https://hub.docker.com/r/hatimalmutairi/lmgaap-maker
minimap2	https://github.com/lh3/minimap2	https://anaconda.org/bioconda/minimap2
MultiQC	https://multiqc.info/	https://anaconda.org/bioconda/multiqc
MUMmer	http://mummer.sourceforge.net/	https://anaconda.org/bioconda/mummer
Pilon	https://github.com/broadinstitute/pilon/wiki	https://anaconda.org/bioconda/pilon
pycoQC	https://pypi.org/project/pycoQC/	https://anaconda.org/bioconda/pycoqc
RaGOO	https://github.com/malonge/RaGOO	https://anaconda.org/imperial-college-research-computing/ragoo
RepeatMasker	http://www.repeatmasker.org/	https://hub.docker.com/r/hatimalmutairi/lmgaap-maker
SAMtools	https://github.com/samtools/samtools	https://anaconda.org/bioconda/samtools
Snakemake	https://snakemake.readthedocs.io/en/stable/	https://anaconda.org/bioconda/snakemake
TEclass	http://www.compgen.uni-muenster.de/tools/teclass/index.hbi?lang = en	https://hub.docker.com/r/hatimalmutairi/teclass-2.1.3b
wordcloud	Not available	https://anaconda.org/conda-forge/wordcloud

### Genome assembly

*De novo* assemblies were performed with Nanopore MinION long reads using Flye^31^. Due to the low quality scores in Nanopore long reads, we mapped high quality Illumina short reads onto the assemblies and created corrected consensus sequences using minimap2^32^ and SAMtools^33^. The consensus sequence was scanned for any contamination or any sequence of vector origin by BLAST+^34^ on the UniVec database^35^. Finally, a polishing step was done to minimise gaps using Pilon^36^.

### Chromosome verification

**Fig. 3:**
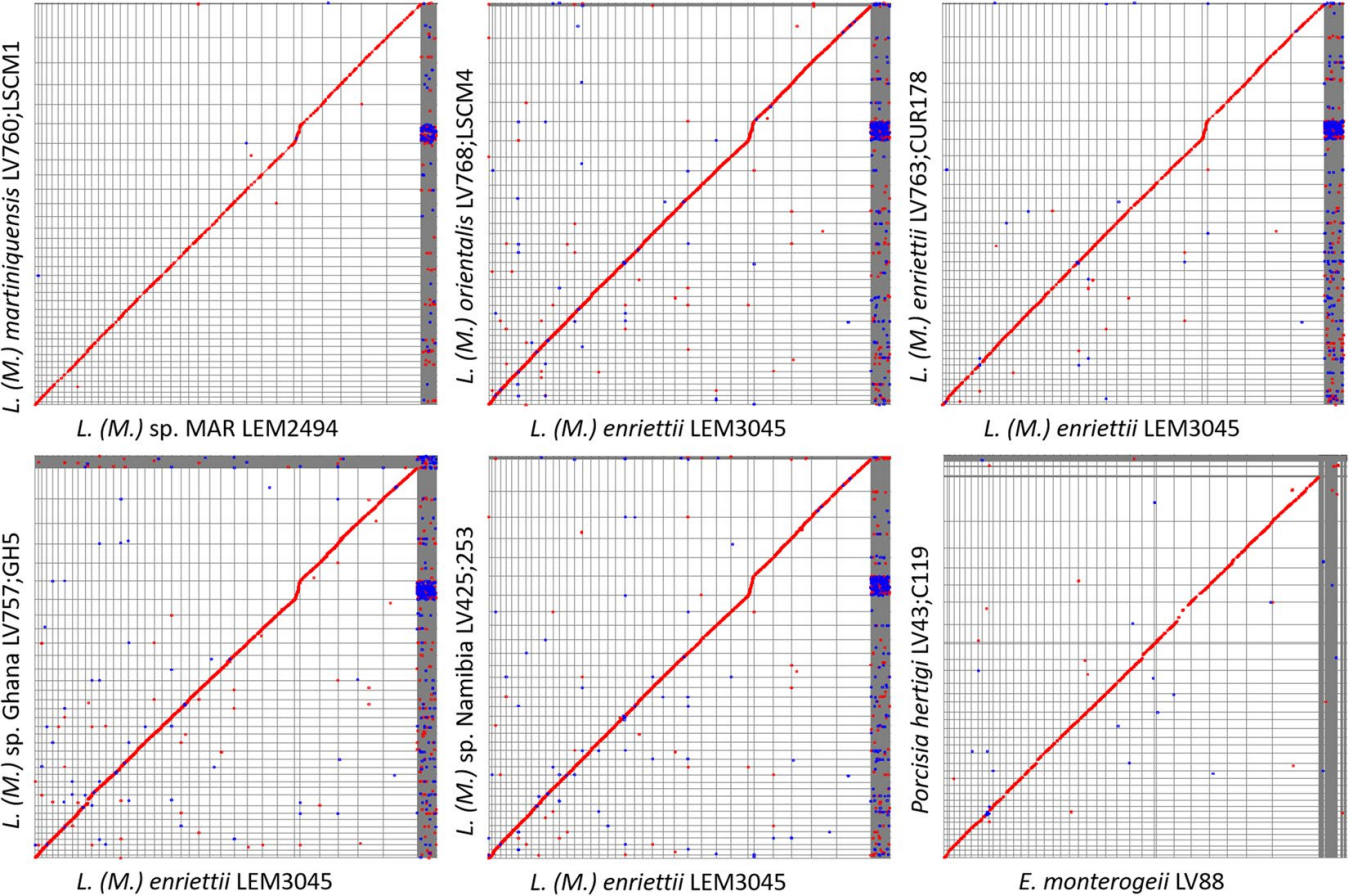
Dotplot representing synteny between each of our genomes and its wordcloud-predicted closest related reference genome, produced using MUMmer.

For all chromosomes of each polished genome, we then ran BLAST + (parameters: -max_target_seqs. 1 -max_hsps 1) against all TriTrypDB^37^ release-47 genomes. The output for each genome was then visualized using wordcloud to suggest the closest relative among TriTrypDB genomes^38^. Then, synteny was plotted for each genome by aligning each of its chromosomes with the corresponding chromosomes of its wordcloud-predicted closest relative, using MUMmer^39^ (Fig. 3). This confirmed that the order and orientation of the chromosomes of each genome was equivalent to those of its closest TriTrypDB genome. Completion was then achieved by sorting and removing any duplicate scaffolds or contigs using funannotate^40^, followed by a final quality check using Genome Assembly Annotation Service (GAAS).

### Repetitive element annotation

**Fig. 4:**
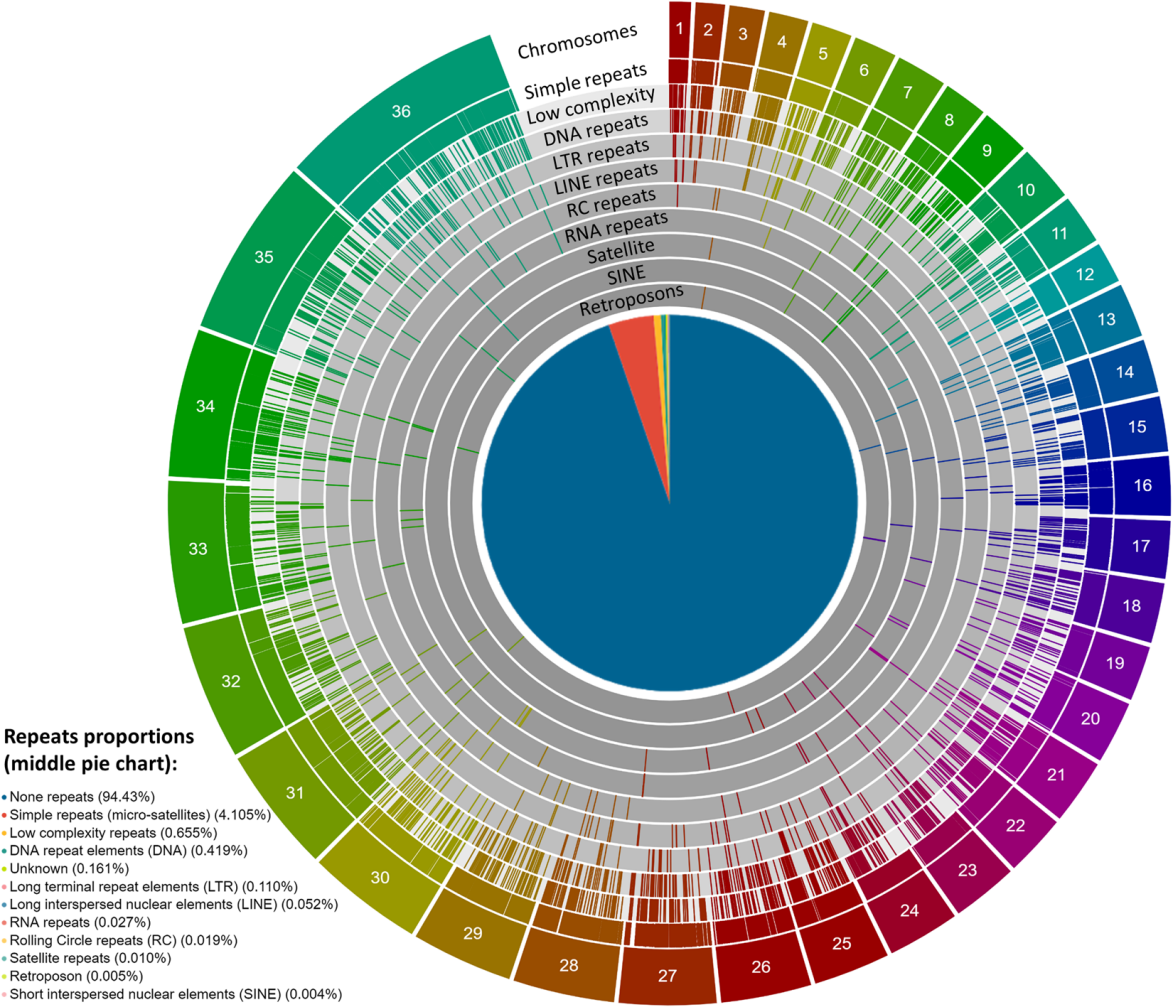
Example genome-wide repeat plot for *L. martiniquensis*, stratified: simple (micro-satellites), low complexity, DNA, long terminal repeats (LTRs), long interspersed nuclear elements (LINEs), RNA, rolling circle (RC), satellites, short interspersed nuclear elements (SINEs) and retroposons. The middle pie chart represent the proportion of each repeat class in the genome: none (94.4%), simple (micro-satellites) (4.11%), low complexity (0.655%), DNA (0.419%), unknown (0.161%), LTRs (0.110%), LINEs (0.052%), RNA (0.027%), RC (0.019%), satellites (0.010%), retroposons (0.005%), SINEs (0.004%).

We identified and classified repeat regions in the polished assemblies using RepeatModeller and TEclass^41^. Then, we generated a stratified genome-wide repeat plot for each assembly^38^ (see also *L. martiniquensis* example in Fig. 4) to assist the decision of which repeats to mask, using RepeatMasker.

### Gene prediction and functional annotation

**Fig. 5:**
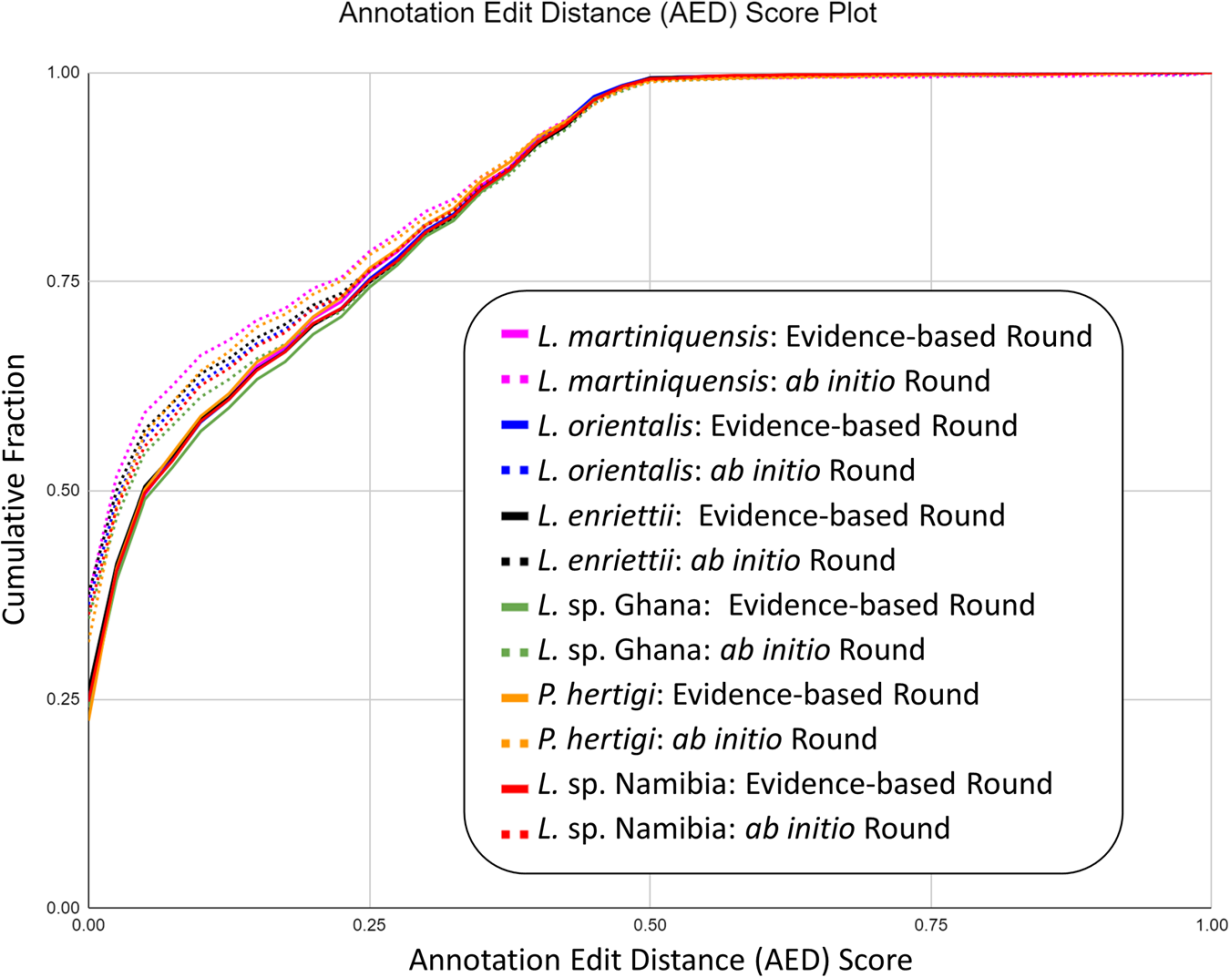
Annotation Edit Distance (AED) score (x-axis) line plot for all assembly annotation rounds: evidence-based (solid line) and *ab initio* (dotted line). Y-axis represents the genome cumulative percentages.

After repeat masking, we annotated the assemblies using the MAKER2^42^ annotation pipeline over two rounds: 1) an evidence-based annotation round using EST, mRNA-seq and protein homology evidence from TriTrypDB release-47 along with our repeat-masking output, 2) an *ab initio* round using AUGUSTUS^43^, with the pre-trained *L. tarentolae* as the model organism. After each round, Annotation Edit Distance (AED) scores were calculated and plotted (Fig. 5). We calculated brief statistics for each round, e.g. the number of genes and other features, using Genometools^44^ and AGAT^45^. After completion of all annotation rounds, we assigned functional annotations from the Uniprot^46^ and Pfam^47^ databases using BLAST + and InterProScan^48^.

### Analysis pipeline

To make sure that all assemblies and annotations are reproducible by future investigators, the entire process from obtaining the SRAs^49–91^ to the annotation assignments^92–97^ has been made available^29^ using Snakemake^98^. This Snakemake pipeline ought to be easily adaptable to the sequencing of further similar parasite genomes, throughout the parasitology community^30^.

## Data Records

Table 3 details the sequencing output. Short and long reads were deposited in the NCBI Sequence Read Archive (SRA)^49–91^. Six BioProjects^23–28^ and six BioSamples^17–22^ were also created at NCBI. The assembled genomes were deposited at NCBI Assembly^99–104^. Additional files containing raw reads quality reports^105–110^, mapped reads^111–116^, classified repeated sequences^117–122^ and functional annotations^92–97^ were deposited at Lancaster University electronic data archive.

**Table 3:** Details of reads, bases and file sizes.

species	Sequencing Platforms	SRA Accession	Number of Reads (GigaReads)	Bases (GigaBase)	File size (Gigabyte)
*L. (Mundinia) martiniquensis*	Illumina HiSeq 4000	SRR13558784	0.783	1.182	2.981
		SRR13558792	1.089	1.644	4.151
	Illumina MiSeq	SRR13558785	0.446	1.327	3.003
	Nanopore MinION	SRR13558786	0.071	3.634	7.323
		SRR13558788	0.006	0.321	0.647
		SRR13558790	0.004	0.468	0.940
		SRR13558793	0.005	0.385	0.774
*L. (Mundinia) orientalis*	Illumina HiSeq 2500	SRR13558774	1.579	1.437	4.843
		SRR13558775	0.618	0.563	1.894
		SRR13558776	1.560	1.420	4.786
		SRR13558777	0.636	0.578	1.947
		SRR13558778	0.735	0.668	2.250
	Illumina HiSeq 4000	SRR13558779	1.079	1.629	4.112
		SRR13558780	1.406	2.123	5.361
	Illumina MiSeq	SRR13558781	0.383	1.135	2.568
	Nanopore MinION	SRR13558782	0.054	3.357	6.756
*L. (Mundinia) enriettii*	Illumina HiSeq 4000	SRR13558795	0.879	1.328	3.350
		SRR13558796	1.214	1.834	4.630
	Illumina MiSeq	SRR13558797	0.506	1.494	3.385
	Nanopore MinION	SRR13558798	0.072	4.365	8.786
*L. (Mundinia)* sp. Ghana	Illumina HiSeq 2500	SRR13558800	1.228	1.117	3.765
		SRR13558801	0.684	0.623	2.096
	Illumina HiSeq 4000	SRR13558802	1.006	1.519	3.833
		SRR13558803	1.407	2.124	5.365
	Illumina MiSeq	SRR13558804	0.520	1.549	3.505
	Nanopore MinION	SRR13558805	0.077	5.390	10.840
*L. (Mundinia)* sp. Namibia	Illumina HiSeq 4000	SRR13558764	0.527	1.567	3.546
		SRR13558765	0.985	1.487	3.753
	Illumina MiSeq	SRR13558766	1.347	2.034	5.136
	Nanopore MinION	SRR13558767	0.068	4.377	8.807
*Porcisia hertigi*	Illumina HiSeq 4000	SRR13558754	0.929	1.403	3.540
		SRR13558755	1.409	2.128	5.374
	Illumina MiSeq	SRR13558756	0.379	1.123	2.541
	Nanopore MinION	SRR13558757	0.019	1.364	2.742
Grand Total			23.708	58.698	139.327

## Technical Validation

### Genomic DNA integrity

Genomic DNA was extracted using Trizol (Invitrogen) and quantified using Qubit® dsDNA HS Assay Kits (ThermoFisher Scientific) prior to sequencing. Concentrations ranged between 68.2 and 120 ng/µL. For consistency, we used the same extracted DNA for all three sequencing platforms (Nanopore MinION, Illumina HiSeq 4000 and MiSeq). Furthermore, we assessed the gDNA high molecular weight using N50 estimates of MinION long reads which were ranged between 12.07 and 22.92 kilobases.

### Contamination screening

We scanned all assemblies for any contamination or any sequence of vector origin by first building a UniVec Database and then using BLAST+ . All contaminants were found either at the beginning or at the end of contigs and then deleted. No contaminants affected assembly integrity.

### Quality of short and long raw sequence reads

We used FastQC to check the sequence quality of Illumina short reads sequences and pycoQC to check the Nanopore long reads sequence quality. We used MultiQC^123^ to output all sequence quality scores in one interactive report^105–110^.

### Assembly validation

Since the analysis took many steps to finish, quality checks were introduced between each step. Some checks were focused on completeness, for instance using BUSCO^124^ as a benchmark for the presence of expected universal single-copy orthologues. Other checks focussed on the correct order and orientation of the chromosomes, for instance MUMmer alignment to find synteny between assemblies and other *Leishmania* genomes. Yet further checks focussed on the accuracy and precision of annotation, for instance using Annotation Edit Distance score (AED) in MAKER2 (Fig. 5). We checked reproducibility of the assemblies and annotations using Snakemake.

## Data Availability

The Snakemake analyses pipeline was deposited at GitHub and Zenodo repositories^29,30^. Links to software used as well as relevant conda and docker containers are given in Table 2.
